# Applicability of anatid and galliform microsatellite markers to the genetic diversity studies of domestic geese (*Anser anser domesticus*) through the genotyping of the endangered zatorska breed

**DOI:** 10.1186/1756-0500-4-65

**Published:** 2011-03-16

**Authors:** Krzysztof Andres, Ewa Kapkowska

**Affiliations:** 1 Department of Poultry and Fur Animal Breeding and Animal Hygiene, Agricultural University of Krakow, Mickiewicza 24/28, 30-059 Kraków, Poland

## Abstract

**Background:**

The lack of a sufficient number of molecular markers seriously limits the cognition of genetic relationships within and between populations of many species. Likewise, the genetic diversity of domestic goose (*Anser anser domesticus*), with a great number of breeds throughout the world, remains poorly understood at the molecular level.

**Findings:**

Thirty-five goose, seventeen duck and eight chicken microsatellite primer pairs were screened for their utility in the cross-species amplification on DNA from 96 individuals of Zatorska breed of domestic geese. Twenty-seven of 42 amplifying primer pairs revealed length-polymorphic products, but three of them were difficult to score. Fifteen primer pairs amplifying the same length product across all individuals. One polymorphic microsatellite locus was assigned by genotyping of known sex individuals to the Z-chromosome.

**Conclusions:**

We present a set of 24 polymorphic microsatellite markers useful for population genetic studies of the domestic goose. Another 15 markers were classified as monomorphic, but they might also be suitable for the assessment of genetic diversity in geese.

## Background

It is currently widely accepted that characterization of genetic diversity of a population at the molecular level provide a great support for decision making in the field of conservation, maintenance and monitoring of genetic resources [1-3]. The main tool in the characterization of the genetic diversity of farm animals is DNA polymorphism analysis of microsatellite loci [4]. This type of analysis was proposed by a joint International Society for Animal Genetics (ISAG) and Food and Agriculture Organization of the United Nations (FAO) working group and used in about 90 percent of all molecular studies of genetic diversity of farm animals [5]. Nonetheless, some of the important domestic species, including domestic geese, are still sparsely genotyped by this method. There are 181 recognized breeds of domestic geese in the world with 158 local populations distributed mainly in Europe and Asia [6]. In 2006, two thirds of distinguished breeds (121) faced the risk of being lost or their risk status was disturbingly unknown. The genetic relationship between a great majority of breeds and the level of genetic diversity of given populations of domestic geese have not yet been revealed. The domestic goose is one of the fourteen major species of domestic animals included in the Global Project for the Maintenance (or Measurement) of Domestic Animal Genetic Diversity [7] but the recommended microsatellite marker list for diversity studies has not yet been proposed for this species. Currently there are known to be only ten species-specific microsatellite markers isolated and evaluated in the graylag (*Anser anser *L.) [8], an ancestor of Western breeds of domestic geese. Furthermore, the effectiveness of panel of 19 [9] or 7 [10] microsatellite markers isolated from swan goose (*Anser cygnoides *L.) was assessed in a biodiversity study of Chinese breeds of domestic geese of swan goose origin and all these markers proved to be useful. It is important to mention that among other species of the genus *Anser*, there is only one single report of two microsatellite markers isolated and tested for amplification in white-fronted geese (*Anser albifrons *L.) [11].

The avian genome appear to be largely conserved and is characterized by a high degree of conserved synteny [12,13]. Thus it has been proved possible to cross-amplify many microsatellite loci in related species, although the probability of cross-species amplifications decreases with increasing genetic distance [14-16]. In the *Anatidae *family, the utilization of primer templates for closely related species were frequently verified as an effective way of obtaining microsatellite loci for various taxa [8,11,17-19], because of the homology of the sequences in the genomes [20]. The application of Canada geese (*Branta canadensis *L.) and chicken microsatellite markers (*Gallus gallus domesticus*) were investigated in the studies of genetic diversity of Chinese domestic goose breeds descended from the swan goose [9,10,21]. Nevertheless, the use of goose and duck microsatellite markers in graylag and domestic geese originated from graylag has been tested sparsely to date [8,22].

The aim of this study was to evaluate the applicability of using selected anatid and galliform microsatellite markers in genetic diversity studies on domestic geese based on Zatorska breed characteristics. The breed was developed between 1956 and 1961 by planned crossing of four Polish local varieties [23]. Three of them (Podkarpacka, Pomorska and Suwalska) share a common ancestor, a graylag goose (*Anser anser*). The fourth variety (Garbonosa) is known to be derived from swan goose (*Anser cygnoides*). The breeders purpose was to create a new breeding stock with better meat and egg production performance and still well adapted to local environmental conditions. Currently a single flock of about 450 animals is maintained as a conservation flock at the Experimental Farm of the Department of Poultry and Fur Animal Breeding and Animal Hygiene of the Agriculture University of Krakow. The breed has been listed in the World Watch List for Domestic Animal Diversity as endangered-maintained due to small population size [23].

## Methods

### DNA sampling and genotyping

In accordance with the breeding program approved for Zatorska geese, every third year we create a breeding stock consisting of 20 mating pens, one male and six females in each (20 males and 120 females in total). The experiment covered 96 individuals from the breeding stock that leave progeny (20 males and 76 females). Individual venous blood samples were taken from the ulnar vein and collected in EDTA spray-coated tubes and stored at -20°C. The amount of blood taken was approximately 2 ml per bird and the final concentration of EDTA in preserved blood was 1.8 mg/ml.

Genomic DNA was extracted from 15 μl of preserved blood. Four hundred microliters of lysis buffer (100 mM NaCl, 10 mM Trizma base, 10 mM EDTA, 2% SDS) was added to the aliquot. The solution was made up to 150 μg/ml with Proteinase K. After overnight incubation at 56°C, the protein was removed by phenol and chlorophorm-isoamyl alcohol extractions and DNA was precipitated by ethanol [24].

Sixty pairs of primers designated for the genotyping of microsatellite loci of five *Anatidae *species (swan goose *Anser cygnoides *L., white-fronted goose *Anser albifrons *Scopoli, Canada goose *Branta canadensis *L., domestic duck, derived from mallard *Anas platyrhynchos *L., harlequin duck *Histrionicus histrionicus *L.) and domestic fowl (*Gallus gallus domesticus*) have been tested on the material of Zatorska geese DNA. The investigated loci were chosen in accordance with previously consulted studies, where the applicability was observed in various goose and duck species. We chose: twenty-one pairs of primers of the CKW set isolated from swan goose [9,10,21], eight markers from the Bca μ set isolated in Canada geese and polymorphic in white-fronted geese [18], eleven domestic duck APH markers of proven usefulness in graylag [22], five TTUCG markers isolated in Canada geese [25], four CAUD-G markers isolated in domestic duck and briefly checked for amplification in domestic geese of European (*A. anser*) and Asiatic (*A. cygnoides*) origin [17], Hhi μ1 and Hhi μ3 markers of harlequin duck [18] and Aal μ1 marker isolated in white fronted geese [11]. Finally, 8 chicken primers pairs (ADL166, ADL210, MCW004, MCW014, MCW085, MCW104, MCW120, MCW264) were chosen according to the reports of their usefulness in the genotyping of Chinese domestic geese [9,10]. Detailed information about each waterfowl and chicken marker are listed in Table 1 and Additional file 1. The sequences of the original clones were checked for duplication using BLASTN v.2.2.4 [26] following Dawson et al. [16] and all the sequences were confirmed to be unique. Additionally, we found out that the sequences of three of the tested microsatellite loci, named CKW12, CKW20 and CKW43 cover the same nucleotide sequence as clones CKW22, CKW24 and CKW410 deposited in the PubMed database, respectively. The optimum annealing temperature for the markers were defined empirically using 12-degree annealing temperature gradient function (between 50 and 65°C) in Mastercycler ep Gradient thermal cycler (Eppendorf), in the preliminary experiment conducted on DNA probes of twenty individuals.

**Table 1:** Characteristics of 24 polymorphic microsatellite loci in Zatorska geese population

Locus	GenBank Accesion No.	*Source species*	Repeat motif of sequenced clone	Primer sequence (5'-3')	Ref.	*T*_a_	*N*	*A*	Allele size range (bp)	*RS*	*PIC*	*H*_O_	*H*_E_	*P*_HWE_	Null allele freq.	*PE*	*PI*	Chromosome, location (bp) (*E*-value)
Aal μ1	U63689	*Anser albifrons*	TG	F: CATGCGTGTTTAAGGGGTAT R: TAAGACTTGCGTGAGGAATA	11	55	96	4	81-91	1.29	0.21	0.16	0.23	0.012	0.151	0.203	0.612	No match
APH12	AJ515893	*Anas platyrhynchos domesticus*	(GAAA)_4_A_2 _(GAAA)_2_	F: TTAGTAGCATGTCAGGTTTATT R: GCTTGTAGACTTCAGAGTTC	22	58	96	2	155-157	1.81	0.35	0.34	0.45	0.033	-0.131	0.265	0.405	Gga2, 130340088 (5.0E-98)
APH13	AJ515894	*Anas platyrhynchos domesticus*	(GA)_10_	F: CAACGAGTGACAATGATAAAA R: CAATGATCTCACTCCCAATAG	22	55	96	2	163-165	1.85	0.35	0.36	0.46	0.027	0.133	0.268	0.398	Gga7, 4082663 (9.0E-56)
APH16	AJ515897	*Anas platyrhynchos domesticus*	(CA)_7_	F: CCTTCTGAACCTTCGTAG R: AAATATAGACTTTTGTCCTGAA	22	58	96	2	144-148	1.94	0.37	0.38	0.48	0.219	0.086	0.277	0.382	Gga4, 36804087 (7.0E-21)
APH20	AJ515901	*Anas platyrhynchos domesticus*	(CA)_9_	F: ACCAGCCTAGCAAGCACTGT R: GAGGCTTTAGGAGAGATTGAAAAA	22	60	96	4	140-150	1.94	0.45	0.48	0.49	0.363	0.006	0.436	0.304	Gga8, 2546006 (3.0E-56)
Bca μ1	AF025889	*Branta canadensis*	(TA)_15 _(CA)_10_	F: TGCTTTTTACCCCCAGTGTTCT R: AGAATCTGCTATATTATTTCCAGCTC	18	61	96	5	115-125	3.81	0.69	0.59	0.74	0.000	0.123	0.677	0.114	Gga12, 4593760 (3.0E-08)
Bca μ5	AF025893	*Branta canadensis*	(CA)_9_	F: AGTGTTTCTTTCATCTCCACAAGC R: AGACCACAATCGGACCACATATTC	18	62	96	2	197-201	1.06	0.05	0.06	0.05	1.000	-0.006	0.051	0.895	Gga1, 74604866 (6.0E-40)
Bca μ6	AF025894	*Branta canadensis*	(CA)_10_	F: TTTAACCCAGTAGCCTATCATGTCA R: GTCTGAAGATAATGCTGCATGGTT	18	60	96	2	141-149	1.18	0.14	0.15	0.16	0.496	0.023	0.127	0.727	Gga2, 55974019 (3.0E-63)
Bca μ7	AF025895	*Branta canadensis*	(CA)_7_N_5 _(CA)_7 _(TTTA)_4_	F: TAGTTTCTATTTGCACCCAATGGAG R: CGGTCCTGTCCTTGTGCTGTAA	18	61	96	2	171-175	1.11	0.10	0.09	0.10	0.225	0.084	0.090	0.812	Gga2, 30816660 (1.0E-14)
Bca μ8	AF025896	*Branta canadensis*	(CA)_8_	F: CCCAAGACTCACAAAACCAGAAAT R:ATGAAAGAAGAGTTAAACGTGTGCAA	18	58	96	4	155-159	2.52	0.52	0.47	0.61	0.003	0.132	0.471	0.238	No match
Bca μ9	AF025897	*Branta canadensis*	(CA)_9_	F: CCCAGTTCCTCTCATTCTCCTT R: AAACAGGGAGGTGAAAGTGCTT	18	61	96	3	104-116	2.03	0.41	0.48	0.51	0.782	0.023	0.339	0.340	Gga7, 22851442 (4.0E-11)
Bca μ10	AF025898	*Branta canadensis*	(CA)_9_	F: ATGTAGCCATGAAAATTAAAAAATG R: CCAGTATTAGCCGAAAAGATGA	18	60	96	2	102-104	1.66	0.32	0.29	0.40	0.008	0.165	0.247	0.441	Gga2, 111179712 (3.0E-09)
CAUD-G007	AY493252	*Anas platyrhynchos domesticus*	(CAG)_5 _(GCA)_5_	F: ACTTCTCTTGTAGGCATGTCA R: CACCTGTTGCTCCTGCTGT	17	61	96	3	116-122	1.32	0.23	0.24	0.25	0.558	0.008	0.217	0.587	No match
CAUD-G012	AY493257	*Anas platyrhynchos domesticus*	(AC)_10_	F: ATTGCCTTTCAGTGGAGTTTC R: CGGCTCTAAACACATGAATG	17	57	96	3	203-211	2.11	0.44	0.53	0.53	0.656	0.006	0.372	0.313	GgaU, NW_ 001477064.1 (1.0E-11)
CAUD-G013	AY493258	*Anas platyrhynchos domesticus*	(AC)_9_	F: ACAATAGATTCCAGATGCTGAA R: ATGTCTGAGTCCTCGGAGC	17	61	96	2	92-98	1.42	0.25	0.32	0.30	0.726	-0.036	0.205	0.542	Gga13, 14041755 (0.009)
CKW5	AY720919	*Anser cygnoides*	AC	F: CAAAGCCCGTCATAGCA R: AAGTTTCGGTCTGGATTGA	21	57	96	2	236-240	1.03	0.03	0.03	0.03	1.000	-0.002	0.031	0.938	Gga3, 50882942 (2.0E-32)
CKW14	AY720927	*Anser cygnoides*	(CCT)_5_	F: AACTGATCCGGCAGAAAACTAA R: ACTTAGCATGCAGCTTCACAAA	9	60	96	2	221-223	1.77	0.34	0.48	0.44	0.348	-0.054	0.260	0.414	Gga17, 4593759 (4.0E-33)
CKW18	AY720929	*Anser cygnoides*	(CAAAA)_7_	F: AATGTGCTGTGTCACATTCTCC R: CATCATCCAACGATTCAGACAT	9	57	96	2	246-250	1.76	0.34	0.41	0.43	0.638	0.022	0.259	0.416	Gga4, 52015193 (1.0E-22)
CKW21	-	*Anser cygnoides*	(TTA)_10_	F: CAAGGTAGTCATAAACCCAGAACA R: ACAAAACTAATGGCAGGAAAC	21	62	96	6	346-375	2.87	0.59	0.45	0.66	0.000	0.193	0.571	0.182	Gga1, 2666911 (2.0E-64)
CKW43	AY790340	*Anser cygnoides*	(CA)_11_	F: TCCAAGGCTTACTTCCCAAG R: CAGAAGACAGGCCTGCAAAT	9	62	20(M)	2	129-131	1.84	0.35	0.68	0.46	0.047	-0.215	-	-	GgaZ, 9308527
							76 (F)	2	129-131	1.94	0.37	0.00	0.49	0.000	0.999	-	-	(0.008)
CKW47	AY790335	*Anser cygnoides*	(T)_8_(TG)_7_	F: AACTTCTGCACCTAAAAACTGTCA R: TGCTGAGGTAACAGGAATTAAAA	9	62	96	2	213-215	1.13	0.11	0.12	0.11	1.000	-0.022	0.099	0.792	Gga4, 68269490 (4.0E-13)
TTUCG1	U66089	*Branta canadensis*	CA	F: CCCTGCTGGTATACCTGA R: GTGTCTACACAACAGC	25,35	58	96	2	113-115	1.32	0.21	0.28	0.24	0.351	-0.072	0.179	0.605	Gga11, 13347684 (1.0E-11)
TTUCG2	U66090	*Branta canadensis*	GT	F: GAGAGCGTTACTCAGCAAA R: TCACTCTGAGCTGCTACAACA	21^1^, 25,35	55	96	2	112-128	1.92	0.36	0.52	0.48	0.518	-0.037	0.275	0.386	No match
TTUCG5	U66093	*Branta canadensis*	TCTAT	F: GGGTGTTTTCCAACTCAG R: CACTTTCCTTACCTCATCTT	21^2^, 25,35	61	96	7	176-216	2.35	0.55	0.55	0.58	0.242	0.012	0.569	0.209	Gga1, 269486597 (6.0E-04)

Ref., References; *T*_a_, annealing temperature; *n*, number of individuals genotyped; *A*, number of alleles; *RS*, allelic richness; *PIC*, polymorphism information content; *H*_O _observed heterozygosity; *H*_E _expected heterozygosity; *P*_HWE_, Hardy-Weinberg equilibrium test *P*-value; *PE*, probability of exclusion; *PI*, probability of identity; M, males; F, females.

^1^under the name WD136

^2^under the name WD206

PCR reactions contained approximately 20 ng of genomic DNA, 20 mM Tris-HCl, 50 mM KCl, 3.0 mM MgCl_2_, dNTP mix (0.2 mM each of dATP, dGTP, dCTP and dTTP), 0.25 U *Taq *polymerase (Invitrogen, Carlsbad, CA) and 400 nM of each primer were carried out in a total volume of 10 μl. The reactions were run on a Mastercycler ep thermal cycler (Eppendorf). PCR cycling conditions were 2 min denaturation at 95°C, followed by 30 cycles of 30 s denaturation at 94°C, 45 s annealing at optimal temperature for given primer (Table 1 and Additional file 1), and 1 min elongation at 72°C. Final elongation took 8 min at 72°C.

PCR products were electrophoresed on 6% denaturing polyacrylamide gels (32 cm × 30 cm × 0.4 mm) by using a Bio-Rad Sequi-Gen GT Nucleic Acid Electrophoresis system (Hercules, CA) run at constant 80 W and 50°C for 3 to 5 hours. The time of electrophoresis was adjusted to optimize the separation with each PCR product length. Gels were silver stained according to methods outlined by Qu et al. [27]. Alleles were distinguished by their mobilities relative to 10 bp DNA Ladder (Invitrogen, Carlsbad, CA).

### Statistical analysis

Mean number of alleles, allelic richness (RS), observed heterozygosity (H_O_), expected heterozygosity (H_E_) assessment of deviation from Hardy-Weinberg equilibrium (HWE), estimated null allele frequency and polymorphism information content (PIC) for each microsatellite locus were determined using Cervus 3.0 computer program [28]. Tests for departures from HWE were performed using a Markov-chain method using the GENEPOP v4.0 [29]. Pairwise tests for linkage disequilibrium based on 7,560 permutations using Bonferroni correction (*P *< 0.05) were performed using FSTAT version 2.9.3 [30]. Sequence similarity between the anatid microsatellites and the chicken genome was determined using the BLASTN program from NCBI, following Dawson et al. [16]. Only matches that scored an Expectation value (*E*) equal or less than 1 × 10^-05 ^were regarded as being significant. The two parents exclusion probability (PE; probability of excluding two putative parents when genotypes of the offspring and both of its parents are known) based on observed allele frequency distributions assuming HWE [31] were calculated in Cervus 3.0 [28]. Combined probability of exclusion [32] over all polymorphic loci was also determined. The probability of identity (PI), as an individual identification estimator, was computed for each locus in Cervus 3.0 [28]. One-way ANOVA and the Tukey's test were applied to examine the possible differences between the mean PIC values of species-specific marker sets using SAS software, version 8.2 (SAS Institute, Cary, NC).

### Statement of Ethical Approval

All procedures performed on the animals were approved by the First Local Ethic Commission for Animal Experiments in Krakow, Poland (Ref. No. 49/2006).

## Results

Altogether, 60 microsatellite primer pairs were tested for amplification and polymorphism on 96 individuals from the Zatorska breed of geese. The amplifications of 42 primer pairs resulted in clear and visible products of the expected size based on the sequenced clone. Among them, 27 loci were polymorphic and 15 were monomorphic (Table 1, Additional file 1). The latter, although not useful in the evaluation of the genetic diversity of Zatorska geese population, may however be helpful in the studies of relationships between various breeds and species of geese. Therefore the information about the monomorphic microsatellite PCR products length and optimal annealing temperature are given in Additional file 1.

The set of successfully amplified microsatellite loci were easy to score on 6% polyacrylamide gels. This was true except for the three loci of poly-A microsatellite repeats (CKW10, CKW11 and CKW12, Additional file 1). The genotyping of the latter may bring difficulties if the 1bp allele size increments affect the occurrence of considerable stutter bands in electrophoresis. The allele size range at a locus of polymorphic markers varied from 2 bp for 5 loci (APH12, Bca μ10, CKW14, CKW43 and TTUCG1) to 40 bp for TTUCG5 locus.

**Figure 1:**
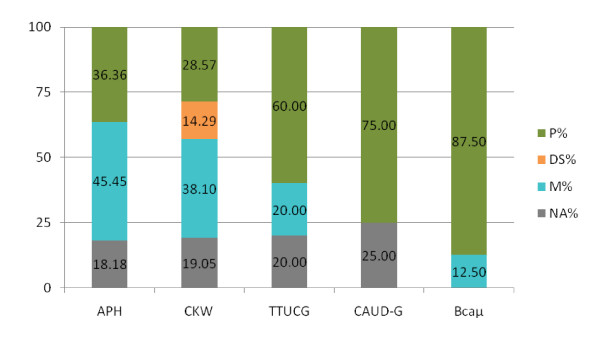
**Proportion of polymorphic (P%), polymorphic but difficult to score (DS%), monomorphic (M%) and non-amplifying (UG%) markers in five marker sets in the study of genetic diversity of Zatorska geese **APH, the set of 11 microsatellite loci isolated in domestic duck (*Anas platyrhynchos domesticus*) [22]; Bca μ, the set of 8 markers from Canada goose (*Branta canadensis*) [18]; CAUD-G, the set of 4 markers from domestic duck (*Anas platyrhynchos domesticus*) [17]; CKW, the set of 21 markers from swan goose (*Anser cygnoides*) [9,21]; TTUCG, the set of 5 markers from Canada goose (*Branta canadensis*) [25,35].

As mentioned earlier, the genetic distance between the source species and the target species affect the cross-utility of microsatellites markers. The genetic distances expressed by DNA - DNA melting temperature (ΔTm) hybridization values between a broad range of bird species has been identified by Sibley and Ahlquist [20]. Unfortunately, those authors have not worked out many species within the *Anatidae *family, but it has been shown that the DNA - DNA TmH among this family is certainly lower than 9.8. Any data for the genus *Anser *are not available there, but the value of genetic distance between this genous and ducks should be close to 6.7 which reflects the distance between *Branta *geese and ducks (genus *Anas*, *Melanitta *and *Aix*). *Branta *and *Anser *are clustered together in many phylogenic trees [33,34]. As has been shown elsewhere [15], the increase of TmH values from 6 to 12 corresponded rapidly to fall in amplification success. Nevertheless, it is hard to predict acurately the probability of amplification of microsatellite loci from various anatid species in domestic geese. The success of amplification of Zatorska geese microsatellite loci with the use of primer pairs designated for the Canada goose (Bca μ and TTUCG marker sets), swan goose (CKW) and pekin duck (APH and CAUD-G marker sets), measured as a proportion of effective primer pairs by every marker set are shown in Figure 1. The sets of markers showed variable usefulness in Zatorska geese diversity studies, but at least three quarters of the markers were effective in each marker set. Among the sets of the markers isolated in Canada geese, the Bca μ primer pairs, pre-verified in other species of genus *Anser *[18], were extremely effective (100%), while TTUCG primers were moderately useful (80%). The largest proportion of polymorphic markers were observed in Bca μ marker set (87.50%) while the APH marker set were the least polymorphic in Zatorska geese studies with 36.36 percent of polymorphic loci and the greatest proportion of monomorphic loci (45.45%). Those sequences were isolated in domestic duck, a species from a subfamily different to geese, which probably suggests that homologous segments are present in the genome of geese but does not behave as hypervariable regions. The application of domestic duck markers of the CAUD-G set was much more effective, but those markers were pre-checked for amplification in geese [17], and 75 percent of them were polymorphic in this study. Only 28.57 percent of primer pairs form the swan goose CKW set amplified polymorphic loci that could be scored reliably. The singular white-fronted goose Aal μ1 marker was highly polymorphic in Zatorska geese study. Two harlequin duck (Hhi μ1, Hhi μ3) markers were ineffective in our studies. Successful amplification and polymorphism of microsatellite loci in Zatorska geese was therefore greater using geese primers than duck primers and is consistent with the phylogenetic relationships among these taxa [33,34]. Applicability was also higher if the chosen primers had been previously tested in domestic or other geese. However, an exception has been observed with swan goose CKW markers, because although they were isolated from close related species, they were poorly polymorphic in Zatorska geese.

No products in appropriate length were found in reactions performed on Zatorska geese genomic DNA with all the primers specific to chicken microsatellite loci. Similarly, an attempt at application of 14 chicken markers in Canada geese ended in failure because none of the chicken PCR primers yielded clean amplifications [35]. Although *Galliformes *and *Anseriformes *are considered sister groups, their genetic distance (TmH) equals 22.9 [20]. It has been shown that a genetic distance larger than 20.0 decreases the probability of amplification of the heterologous microsatellite loci to 20% [14]. Furthermore, the value of sequence divergence above around 10-15 TmH often is too high for cross-species amplification of microsatellite sequences [14]. Therefore the distance between chicken and waterfowl is believed to be large enough to inhibit the amplification of many chicken loci in Zatorska geese. Surprisingly, in the studies of biodiversity of Chinese native geese breeds, the usefulness of eight chicken microsatellite markers which were tested in our experiment was stated [9,10].

Four primer pairs tested in this study (Aal μ1, APH12, APH19, Hhi μ1) were previously examined in the graylag goose [8] providing polymorphic products. Three of them, Aal μ1, APH12 and APH19 were effective in Zatorska geese. We obtained one allele more in Aal μ1 marker than in the graylag, whereas in the APH19 locus we found single allele in Zatorska geese. Two alleles were found in APH12, both in Zatorska geese and in graylag [8]. To assess the suitability of the microsatellite markers in domestic geese diversity studies, we examined the presence of linkage between the polymorphic loci. There was no significant evidence of linkage disequilibrium after Bonferroni correction in any pairwise comparisons of the polymorphic microsatellite markers [see Additional file 2]. At the moment, all of the microsatellite markers isolated or adapted for the studies in domestic geese are anonymous and are not assigned to chromosomes of this species, but their putative genomic locations can be predicted based on sequence homology to the assembled chicken (*Gallus gallus domesticus*) or the zebra finch (*Taeniopygia guttata*) genome [13,16]. Sixteen of the 24 polymorphic loci were assigned by Blast analysis to a predicted chromosomal location on the chicken genome based on sequence homology (with E-values ranging between 3 × 10^-08 ^to 5 × 10^-98^). One further domestic duck sequence (CAUD-G012) showed homology to existing chicken genomic sequence of unknown location. We demonstrate that these markers are distributed relatively wide over 9 chromosomes (Table 1). Also, on the basis of the results of linkage analysis it is highly likely that most of the loci can be broadly distributed on different chromosomes, and thus may also be potential markers of multiple major genes associated with economically important traits. Some of the microsatellite loci, which seems to be putative orthologous between domestic geese and duck are already located in the duck chromosomes by genetic linkage mapping [36]. Among duck markers which were polymorphic in Zatorska geese studies, the CAUD(G)-007 and APH12 markers are placed on 1^st ^and 2^nd ^large duck chromosomes, respectively [36]. Moreover, three of the APH markers (APH8, APH12, APH19), used in our study, have been identified as flanking markers of QTLs in the duck genome. The APH08 locus was considered as a flanking marker of QTLs influencing body weight at six weeks of age and the weight of the heart [37]. The last trait was also linked to APH12 in another linkage group. Marker APH19 was linked to QTL that affects both the body weight of ducks at two weeks of age and girth of shank [37].

A total of 69 distinct alleles were found among the 24 polymorphic and easy to score microsatellite loci examined in the Zatorska geese breeding population (Table 2). Eight of the polymorphic loci displayed a significant deviation from HWE (*P *> 0.05) and also high predicted null allele frequency (equal or above 0.1). In the CKW43 locus a different distribution of alleles in male and female were identified. There was no case of a heterozygous state in the CKW43 locus in females (N = 76), therefore the observed heterozygosity (H_O_) equals zero. Inversely, in 20 males the value of H_O _assumed 0.68 and was greater than the expected heterozygosity (H_E _= 0.46). Such a situation indicate that the CKW43 locus is Z-chromosome linked, therefore information about distribution of alleles in the CKW43 marker in females was excluded from calculations of genetic variability in the population. Upon Blast analysis, the original sequence of the swan goose CKW43 clone was assigned a location on chicken Z-chromosome. The length of the homologous sequence was only 36 base pairs (89% of sequence similarity), thus the significance of the match was low (*E *= 0.008, Table 1). This is the first report, to the authors' knowledge, concerning Z-linked microsatellite marker in the *Anser *genus. However three Z-linked loci has been identified in related species such as Canada goose (*Branta canadensis*) [18] and light-bellied Brent goose (*Branta bernicla hrota*) [38].

**Table 2:** Characteristics of five microsatellite marker sets in Zatorska geese

Marker set	Amplifying markers (Polymorphic markers)	Percent of polymorphic markers of those amplifying	MNA	St. Dev	RS	St. Dev	PIC	St. Dev
APH	9 (4)	44.4	2.50	±1.00	1.89	±0.07	0.38	±0.05
Bca μ	8(7)	87.5	2.86	±1.21	1.91	±1.00	0.32	±0.24
CAUD-G	3(3)	100	2.67	±0.58	1.62	±0.43	0.31	±0.09
CKW	17(9)	52.9	2.67	±1.63	1.73	±0.66	0.29	±0.20
TTUCG	4(3)	75.0	3.67	±2.89	1.86	±0.52	0.37	±0.17

Mean			2.79	±1.38				

MNA, mean number of alleles in polymorphic markers;

RS, mean value of allele richness;

PIC, mean polymorphic information content index

One of the main measures of the suitability of microsatellite markers to assess genetic diversity in the population is the mean number of alleles at a given locus [17]. The average number of alleles per locus (±St. Dev) among 24 polymorphic markers in Zatorska geese was 2.85 ± 1.42. According to Barker [39], each microsatellite should exhibit at least four alleles to be considered useful in the evaluation of genetic diversity in order to reduce the standard errors of distance estimates. Only six of our studied markers fall within this presumption. This represents 22 percent of the polymorphic markers, but it is worth emphasizing that only one breed with small population was covered in the experiment. Such conditions may indicate for inbreeding, but systematic attempts have always been made to maintain the genetic variation and large effective population size of the Zatorska geese flock. To reduce the rate of inbreeding, mating of close relatives were avoided, so the average level of inbreeding in the population remained low (below 4%) with less than 0.1% increase per generation [40]. In order to determine the applicability of the markers we also calculated the mean number of alleles across polymorphic loci (MNA) in five species-specific marker sets separately. The highest values of MNA was observed in the TTUCG markers (3.67) and the lowest MNA value was in the APH marker set (2.50; Table 2). The mean number of alleles across loci isolated in Canada and swan geese was relatively high. The results also confirmed the usefulness of markers from the CAUD-G set, which were isolated in the domestic duck but rated as effective in domestic geese studies [17].

Estimates for within-population diversity parameters computed on the base of the results of genotyping of 24 polymorphic microsatellites are shown in Table 1. The mean allelic richness index ranged between 1.03 (CKW5) and 3.81 (Bca μ1). The lowest and greatest polymorphism information content (PIC) per locus was 0.03 (CKW5) and 0.69 (Bca μ1), respectively. An average observed heterozygosity (H_O _± St. Dev) in a population was 0.35 ± 0.18 and ranged from 0.03 to 0.59 in CKW5 and Bca μ1 markers, respectively. The combined value of H_E _over all polymorphic loci was 0.38 ± 0.20. The two-parent probability of exclusion (PE) depended greatly on markers and ranged from 0.03 (CKW5) to 0.67 (Bca μ1). Combined PE value for all polymorphic loci was 99.98%. The overall probability of identity (PI), considering all microsatellite markers, equals 2.79 × 10^-09^. Both the values of combined PE and PI obtained in Zatorska geese allow us to ascertain that the use of tested polymorphic markers enable to determine not only the overall level of genetic variability of the population, but also the relationships between individuals. This information is particularly important in breeding work in such a small population in which the maximum of the existing genetic variability should be maintained.

Ranks of best suitable markers for domestic goose diversity studies are designed on the basis of the values of RS and PIC indexes. The average RS and PIC values per marker set are summarized in Table 2. The main values of PIC in five species-specific marker sets ranged from 0.29 in CKW markers to 0.38 in APH and no statistically significant differences (*P *< 0.05) between main PIC values were found. The detailed values of RS and PIC for each polymorphic marker are given in Table 1. Half of the six markers of RS index value amounting to over 2.0 belong to the Bca μ marker set. The next most common set in this ranking was CKW with two markers. Four of the six most allelic richness loci are also characterized by the PIC index values greater than 0.5.

The levels of PIC or RS indexes are frequently considered indicators of suitability of markers in conservation biology studies. Regardless of the species specificity, it is accepted, that the most useful markers in the genetic diversity studies are those of PIC > 0.5 [41]. Moderately informative markers are these of midrange polymorphism (0.25 <PIC > 0.5), and low informative are these when PIC < 0.25 [17,42]. The PIC value exceeded 0.25 at 15 loci in Zatorska geese (Table 2), which could provide enough information for the assessment of genetic diversity in just this one breed. In the experiment carried out on the 26 Chinese geese breeds [9], 13 of the 31 markers were moderately informative, but the PIC values of the remaining markers exceed 0.5. Comparison of the mean values of PIC for different series of polymorphic microsatellite loci in Zatorska geese showed no statistically significant differences, although these loci have been isolated in different taxa. The values of PIC or heterozygosity estimates calculated from microsatellite markers in domestic birds are usually much lower than in their wild ancestors. In this context it is interesting that the values of H_O _and H_E _estimated from polymorphic loci in the wild graylag amounted 0.36 and 0.42, respectively [8], and are almost identical to the mean values obtained in domestic Zatorska geese. Therefore the proposed set of polymorphic markers can be considered as sufficient for detailed studies on the population structure. It seems reasonable to suppose that these markers, which are adapted from other species, do not affect the undervaluation of genetic parameters.

Recently, 37 new microsatellite sequences have been isolated and classified as polymorphic in light-bellied Brent goose (*Branta bernicla hrota*) [38] that can be utilized for other goose studies, especially if the sequences are checked to be conserved between Brent goose and chicken or zebra finch. New advances have also been made to create conserved microsatellite primer sets that are able to amplify across a wide range of bird species [43]. The method consists of selecting those EST microsatellite loci that had a high degree of sequence homology of primer bind sites between two genetically distantly related bird species, the zebra finch and the chicken, for which genomes assemblies are available. This method could be applied to adopt microsatellite markers for domestic geese, as well as other species.

Regardless of the origin of the markers, we identified ineffective, monomorphic and polymorphic markers with a varying number of alleles in sets of markers isolated from swan goose, Canada goose or domestic duck. These results are consistent with the results of other authors, both in relation to the loci isolated in the species [8] and markers adapted from related species [44]. Admittedly a decline in the effectiveness of microsatellite markers isolated from more phylogenetically distant taxa as chicken and harlequin duck has been observed.

Considering the panel of 24 polymorphic markers separately, it can be concluded that they constitute a useful set of markers for the study of genetic relationships between individuals in a given population, as well as for the genetic diversity studies of domestic geese, both to the sufficient number of markers and their potential high level of variability.

## Competing interests

The authors declare that they have no competing interests.

## Authors' contributions

KA and EK contributed to the design of the experiment and prepared the manuscript. KA performed the polymorphic amplification of the microsatellite primers in Zatorska geese and conducted the statistical analysis of the data. All authors read and approved the final manuscript.

## Supplementary Material

Additional file 1 **Description of microsatellite primer pairs with no utility for Zatorska geese biodiversity studies**. The table presents the species in which the markers were isolated, primer sequences, references, annealing temperatures if amplifying, number of alleles in Zatorska geese and their size range if polymorphic. Click here for file

Additional file 2 ***P*-values of linkage disequilibrium test between microsatellite loci pairs in Zatorska geese**. The pairwise tests for linkage disequilibrium were performed for all possible pairwise comparisons of the sampled polymorphic loci. The significant *P*-values are in bold characters. No *P*-values that remained significant after Bonferroni correction have been indicated. Click here for file
